# Early root migration after a mandibular third molar coronectomy

**DOI:** 10.1007/s10006-022-01072-z

**Published:** 2022-05-21

**Authors:** Rashida N. Simons, Jacco G. Tuk, Jean-Pierre T. F. Ho, Naichuan Su, Jerome A. Lindeboom

**Affiliations:** 1 Department of Oral and Maxillofacial Surgery, Amsterdam University Medical Center and Amstelland Hospital, University of Amsterdam, Meibergdreef 9, 1105 AZ Amsterdam, the Netherlands; 2 Departments of Oral and Maxillofacial Surgery, Amsterdam University Medical Centers and Northwest Clinics, University of Amsterdam, Amsterdam, the Netherlands; 3 Department of Oral Public Health, Academic Centre for Dentistry Amsterdam (ACTA), University of Amsterdam and Vrije Universiteit Amsterdam, Amsterdam, The Netherlands

**Keywords:** Coronectomy, Third molars, Root migration, Mandibular third molar

## Abstract

**Purpose:**

This prospective cohort study aimed to assess early root migration after a coronectomy of the mandibular third molar at 2 and 6 months after surgery.

**Methods:**

We included all patients treated with a coronectomy of an impacted mandibular third molar. The primary outcome measure was the extent of postoperative root migration after 2 and 6 months. Migration was measured as the distance between the root complex and a fixed point on the inferior alveolar canal. The secondary aim was to identify factors (age, impaction pattern, and patient sex) that affected the extent of root migration.

**Results:**

One hundred and sixty-five coronectomies were performed in 141 patients (96 females and 45 males; mean age 33.1 years, SD 16.0). The 2-month checkup was completed by 121 patients that received 141 coronectomies. The 6-month check-up was completed by 73 patients that received 80 coronectomies. The mean root migrations were 3.30 mm (SD 2.53 mm) at 2 months and 5.27 mm (SD 3.14 mm) at 6 months. In the 2–6-month interval, the mean root migration was 2.58 mm (SD 2.07 mm). The extents of migration were similar during the 0–2-month interval and the 2–6-month interval (*p* = *0.529*). Younger age was associated with greater root migration, and females experienced significantly greater migrations than males (*p* = *0.002*).

**Conclusion:**

Roots migrated more rapidly in the first two postoperative months, compared to the 2–6-month interval. Age was negatively correlated with the extent of root migration, and females showed significantly greater migrations than males.

## Introduction

The surgical removal of mandibular third molars is a common procedure. The removal of a lower third molar can potentially cause permanent neurosensory disturbances [1].

A coronectomy is a successful alternative procedure to reduce the risk of inferior alveolar nerve (IAN) damage, when lower third molar roots are in close proximity to the IAN [1, 2]. It involves partial removal of the mandibular third molar; the crown is removed, but the root complex remains intact in the alveolar bone.

The prevalence of IAN injuries after a total mandibular third molar removal is between 0.35% and 8.4% [3, 4]. IAN injuries can cause hypoesthesia, paresthesia, hyperesthesia, or dysesthesia of the lower lip, chin, and gum area. Most (96%) IAN injuries are temporary, and patients recover within the first 4–8 postoperative months [3]. The prevalence of permanent IAN injury is 0.0%–2.1% [5–10]

It has been demonstrated that 84%–97% of root complexes show signs of migration after a coronectomy [5, 6, 11]. Root migration primarily occurs within the first year after the procedure [2, 6, 12], and it seems to plateau 24 months after the procedure [11, 12].

One factor that affects the extent of root migration is age. Several studies have shown that migration slows down or stops as patient age increases [5, 11, 13, 14]. A second factor that affected the extent of root migration is the patient sex. Leung and Cheung [2] and Kohara et al. [13] found that roots migrated to a greater extent in females than males. Contrary, in another study from Leung and Cheung [11] and Yeung et al. [14] no significant difference in the extent of migration between males and females was observed. Studies have shown that other factors, including root morphology, impaction pattern, impaction depth, and eruption status, did not have significant effects on root migration [2, 6, 11, 13].

Root migration can result in root complex eruption in the oral cavity and can cause sensitivity to cold and discomfort in the exposed root area [2, 7]. This complication is one of the most common reasons for retreatment to remove the root complex [2, 5–7, 12, 13]. However, the prevalence of root eruption is low [2, 5–7]. Removing the root rarely causes an IAN injury because the root complex mostly migrates away from the IAN [2, 6, 7]. This migration reduces the risk of an IAN injury during the removal procedure of the root complex [2, 6, 7].

Migration of the root complex can also result in contact between the root complex of the mandibular third molar and the adjacent second molar [6, 15]. Pedersen et al. [6] found that, after a coronectomy, 24.4% of root complexes followed this pattern of migration. Moreover, this pattern was observed in vertically, horizontally, and mesio-angulated impacted root complexes [6].

Most previous studies that investigated coronectomies and root migrations evaluated the extent of root migration at 6 months after the procedure [5, 7, 11]. The aim of the present study was to assess early root migration of the root complex after a coronectomy of the mandibular third molar and find more detailed knowledge about the speed and distance of the root migration complex, measured by performing panoramic imaging at 2 and 6 months postoperatively. Our secondary aim was to identify factors (age, sex, and impaction pattern) that might influence the extent of root migration.

## Materials and methods

This article is reported according to the STROBE (Strengthening the reporting of observational studies in epidemiology) guidelines (strobe-statement.org).

### Ethical considerations

This prospective study was reviewed and approved by the institutional Medical Ethics Committee of Amsterdam University Medical Center (reference number W21_264). The study was conducted in accordance with Good Clinical Practices and the Declaration of Helsinki, as amended in Somerset West, Republic of South Africa, in 1996. Patients were provided with information to explain the study, and all patients consented to participate in the study. Patients also agreed to attend all three appointments (the surgery and two control visits).

### Eligible patients

The study was conducted between January 2019 and December 2020, and patients were eligible for this study when they were referred to the Oral and Maxillofacial Surgery department of Amstelland Hospital for surgical removal of an impacted mandibular third molar. A coronectomy was considered, when one or more of the following radiographic signs was observed on the orthopantomogram (X-OPT) images (Orthopantomograph® OP100 D, GE Healthcare, Dental, Tuusula, Finland) [11]:

1. Darkening of the roots
2. Narrowing of one or both white lines that represented the inferior alveolar canal
3. Narrowing of the root
4. Interruption and/or loss of one or both white lines that represented the inferior alveolar canal
5. Diversion of the IAN

When a patient showed one or more of these radiographic signs, a computed tomography (CT) scan (Philips Ingenuity 128 CT Scanner, Integrity Medical, Fort Myers, Florida, USA) was performed to assess the proximity of the third molar roots to the IAN. When the CT scan confirmed that the roots were close to the IAN, a coronectomy was performed.

Patients were included in the study; they were aged 18 years or older.

Exclusion criteria were:

1. No signs of proximity to the inferior alveolar canal on X-OPT or CT-scan images
2. Patients aged below 18 years old
3. Deep carious lesions in the mandibular third molar
4. Patients suffering from systemic diseases, predisposing them to infection
5. Patients that failed to show up for follow-up appointments

### Outcome measure

The primary outcome measure of this study was the extent of root migration within the first postoperative 6 months.

### Surgical technique

Coronectomy of the impacted mandibular third molar was performed with the patient under local anesthesia. All surgeries were performed by two oral and maxillofacial surgeons—experienced in performing coronectomy—in a standardized fashion, and a similar technique was used in all cases.

**Fig. 1:**
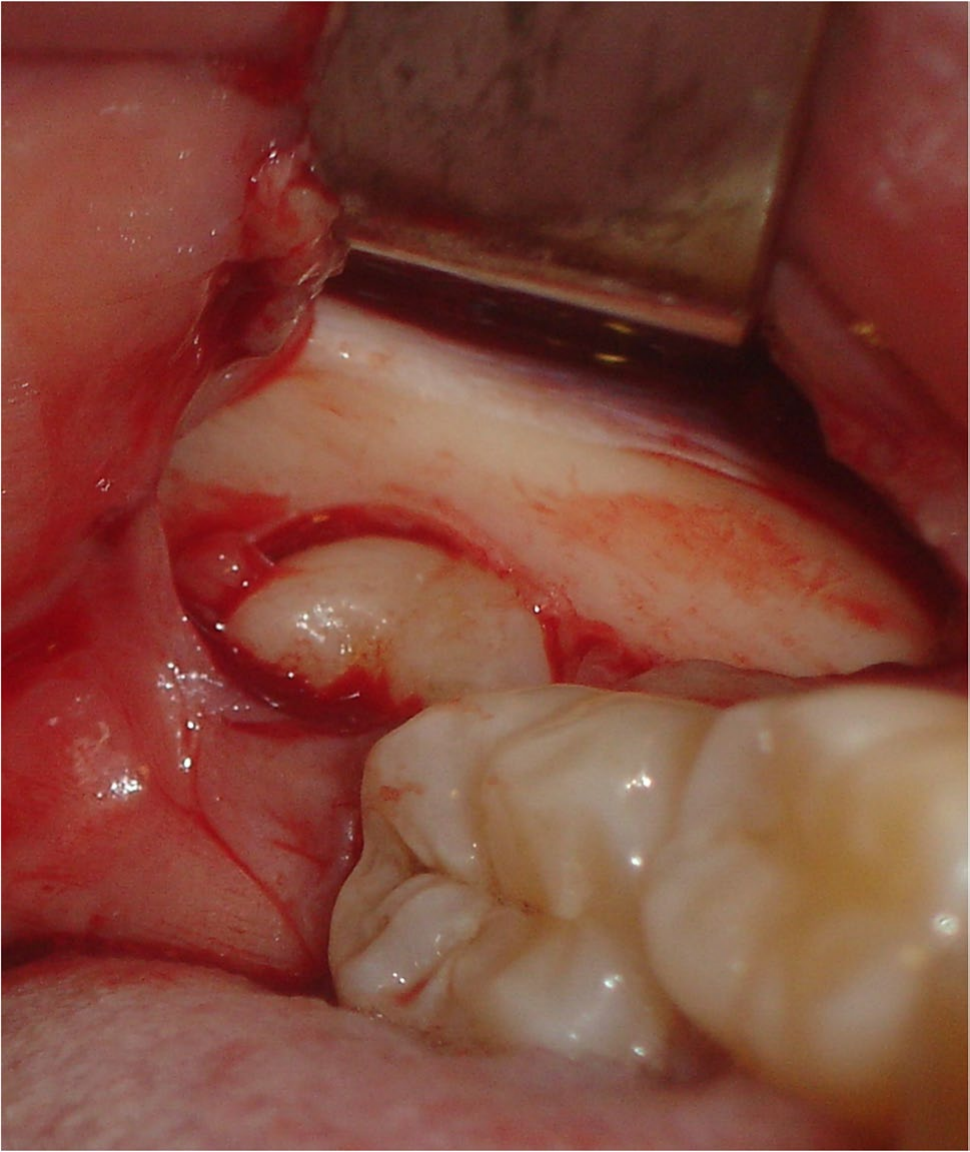
Exposure of the crown of the mandibular third molar after reflection of the triangular flap

**Fig. 2:**
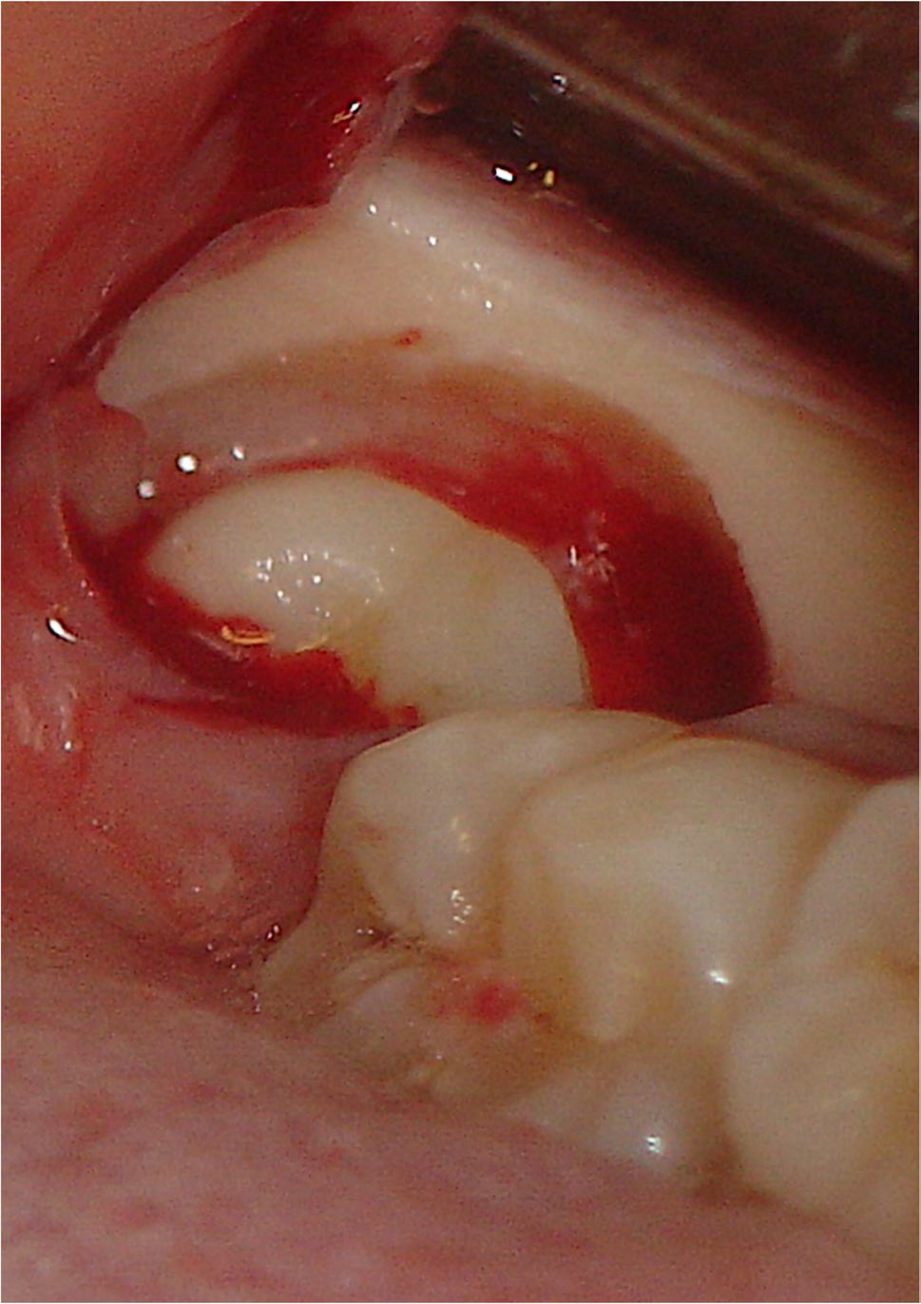
Bone was removed with a steel round surgical bur to expose the cementoenamel junction of the mandibular third molar

**Fig. 3:**
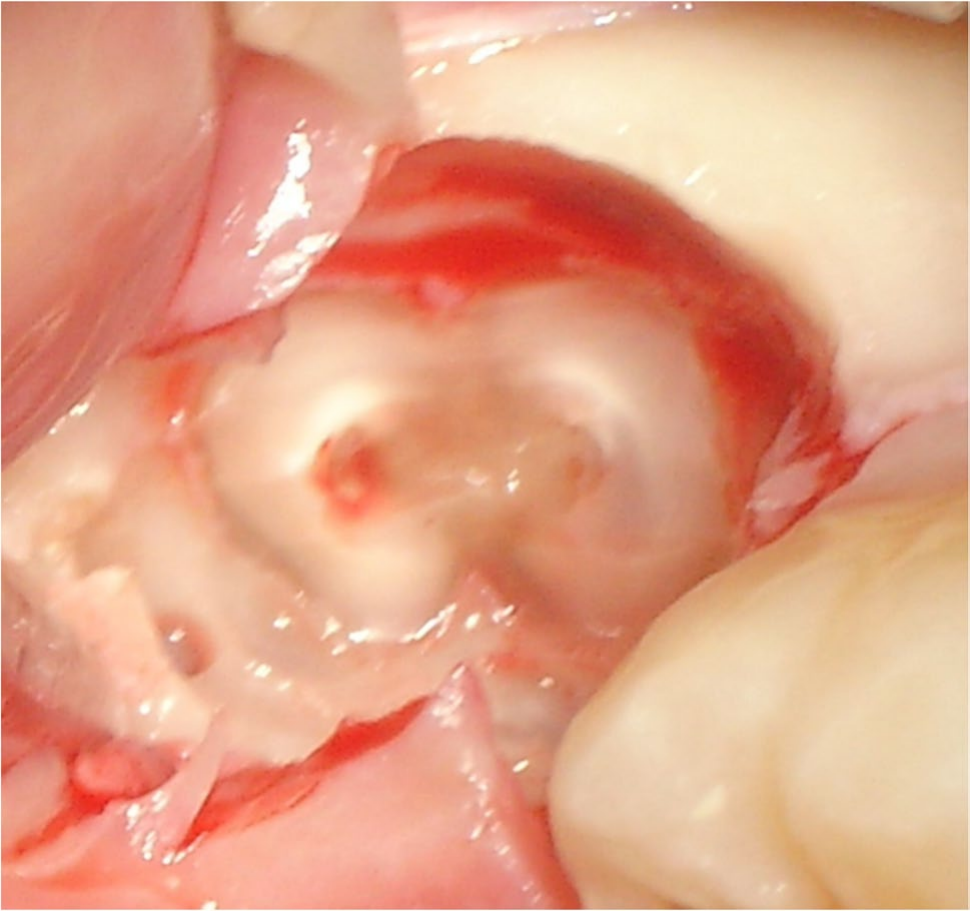
The roots after the completed coronectomy shortened to 4 mm below the bony margin

All patients received a standardized, mandibular nerve block injection, with additional local infiltration of the buccal nerve. The temperature, type, and amount of anesthetic (40 mg articaine/hydrochloride with 0.01 mg epinephrine, administered with a 1.7-mL syringe, Ultracain D-S forte; Sanofi-Aventis, Netherlands BV, Gouda, the Netherlands), the type of needle (27 gauge/0.40 × 35 mm), and the location of administration were all standardized according to the hospital protocol. A triangular flap incision was used in all coronectomies. Briefly, an incision was started at the distobuccal edge of the second molar, which was continued at a slightly oblique angle, and then curved forward into the mandibular vestibule. The second part of the incision started from the mandibular ramus and ended at the distobuccal aspect of the second molar. After exposure of the crown (Fig. 1), any bone overlying the crown of the impacted third molar was removed with a steel round surgical bur exposing the cementoenamel junction of the tooth. (Fig. 2) Next, a steel fissure surgical bur was used to separate the crown from the roots. The root was shortened to 3–4 mm below the bony margin and checked for mobility (Fig. 3). Copious irrigation with sterile saline was performed with rotary instrumentation. Dental follicular soft tissue was removed, and the socket was thoroughly irrigated with saline. The surgical site was primarily closed with 3/0 Undyed Vicryl Rapide (Ethicon, Somerville, MA, USA).

After the procedure, patients were prescribed ibuprofen as an analgesic and a chlorhexidine mouthwash (0,12%) for 5 days.

### Measuring techniques

**Fig. 4:**
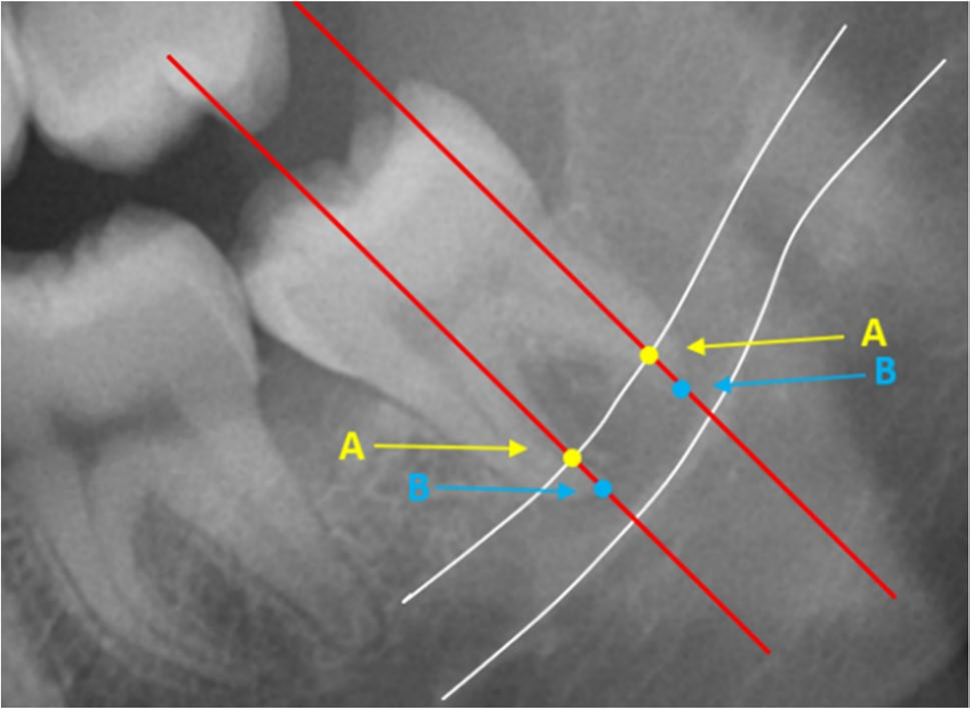
Measuring technique 1: The distance between the roots and the inferior alveolar canal (white lines). Point A: intersection between the long axis of the molar and the upper white line of the inferior alveolar canal; point B: intersection between the long axis of the molar (red lines) and the apices of the mesial and distal roots

**Fig. 5:**
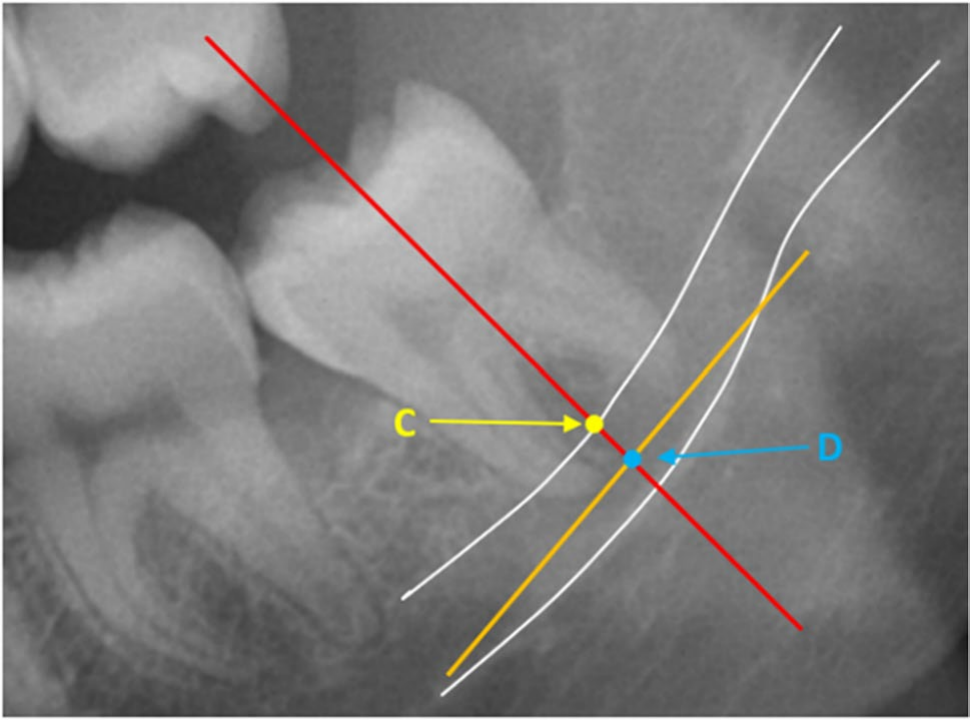
Measuring technique 2: The distance between a line tangent to the two roots (orange line) and the inferior alveolar canal (white lines). Point C: intersection between the long axis of the molar and the upper white line of the inferior alveolar canal; and point D: intersection of the tangent line and the long axis of the molar (red line)

**Fig. 6:**
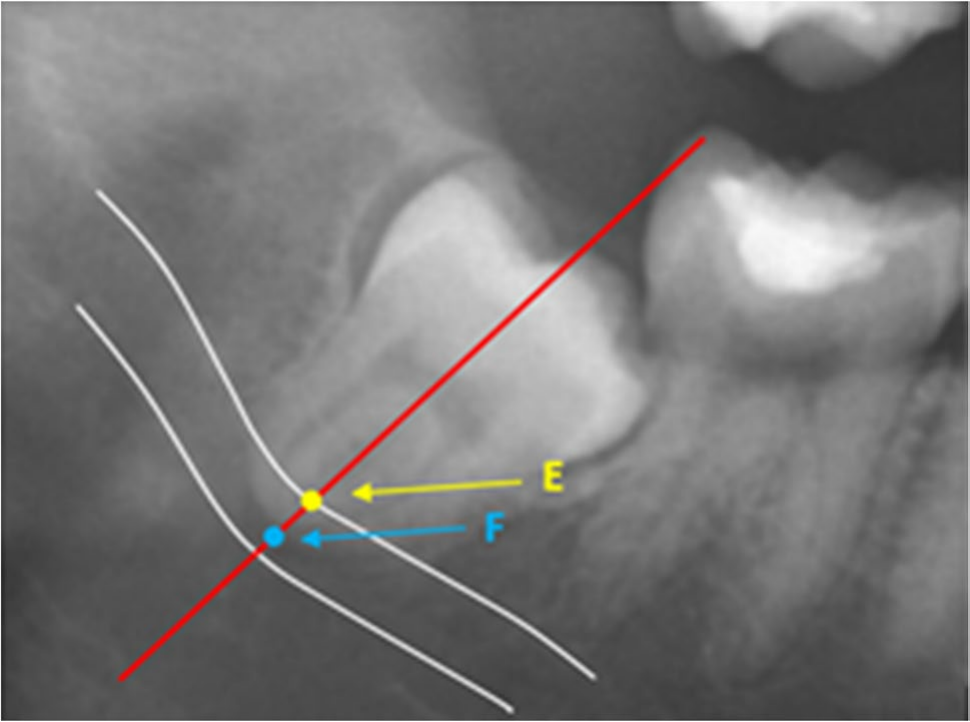
Measuring technique 3: The distance between fused roots and the inferior alveolar canal (white lines). Point E: intersection between the long axis of the molar (red line) and the upper white line of the inferior alveolar canal; and point F: intersection between the long axis of the molar and the apex of the root

In this study, root migration was measured with three different techniques, depending on the root form. In all techniques, migration was measured as the distance between the root and a fixed reference point on the inferior alveolar canal. All measurements were taken with VisiQuick 3.0.1.819 software (VisiQuick 3.0.1.819, Citodent Imaging, Amsterdam, The Netherlands) on panoramic images (Figs. 4–6).

Measuring technique 1 (Fig. 4) was applied to lower third molars with two separate root apices (mesial and distal). In this method, two lines were drawn over the X-OPT image. These lines illustrated the long axis of the third molar and were centered over the mesial and distal apices, one for each apex. On each line, two points were identified: Point A was the intersection between the long axis of the upper white line of the inferior alveolar canal; point B was the intersection between the long axis of the third molar and apex of the root. The distance between points A and B was measured for each root apex. With this method, the root migration of the mesial and the distal roots could be assessed separately.

The second measuring technique (Fig. 5) was also applied to lower third molars with two separate apices. Two lines were drawn on the X-OPT (panoramic) images: one line illustrated the long axis of the third molar, and the other line was tangent to the mesial and the distal root apices. Again, two points were identified: point C was the intersection of the long axis of the root and the upper white line of the inferior alveolar canal, and point D was the intersection of the long axis of the third molar with the line tangent to the mesial and distal root apices. The distance between points C and D was measured.

The third technique (Fig. 6) was applied to lower third molars with fused roots. For this technique, only one line was drawn: the long axis of the third molar. On this line, two points were identified: point E was the intersection between the long axis of the third molar and the upper white line of the inferior alveolar canal, and point F was the intersection between the long axis of the third molar and the apex of the fused root. The distance between points E and F was measured.

The measurements from technique 1 were divided into two groups: mesial root measurements and distal root measurements. The measurements from techniques 2 (tangent measurement) and 3 (measurement of fused roots) were combined into one group; this was the main study group for this study. Therefore, we analyzed the following three study groups:

1. Main study group: measurements for all third molars with a line tangent to two separate apices and all fused root measurements
2. Mesial root measurement study group
3. Distal root measurement study group

All measurements were corrected for a 1.3 × magnification applied in the panoramic images.

### Identification of influencing factors

We investigated whether the patient age, sex, or impaction pattern might affect the extent of root migration.

Patients were divided into five age groups (18–21, 22–25, 26–30, 31–40, and 40 + years).

Impaction patterns were classified to three different categories [16]:

1. Inclination of the third molar, categorized as mesial, vertical, or distal
2. Impaction depth, categorized as follows:

- A = The highest portion of the third molar was above the occlusal plane of the second molar
- B = The highest portion of the third molar was between the occlusal plane and cement–enamel junction of the second molar

- C = The highest portion of the third molar was below the cement–enamel junction of the second molar

3. The space between the distal part of the second molar and the ramus of the mandible is categorized as follows:

- I = Sufficient space to accommodate the mesio-distal diameter of the third molar.
- II = Insufficient space to accommodate the mesio-distal diameter of the third molar.
- III = Nearly all the third molar is in the mandible.

### Statistical analyses

The one-way ANOVA test was performed to determine whether the age or impaction pattern influenced the extent of root migration (α = 5%). The two-way ANOVA test was performed to determine whether patient sex influenced the extent of root migration over time.

## Results

Patient information such as demographic characteristics, age, and sex was recorded prior to the operation. Panoramic radiographs were performed preoperatively, immediate postoperatively, and at the 2- and 6-month follow-up appointments. Patients that did not attend the follow-up appointments were excluded from the analysis.

**Fig. 7:**
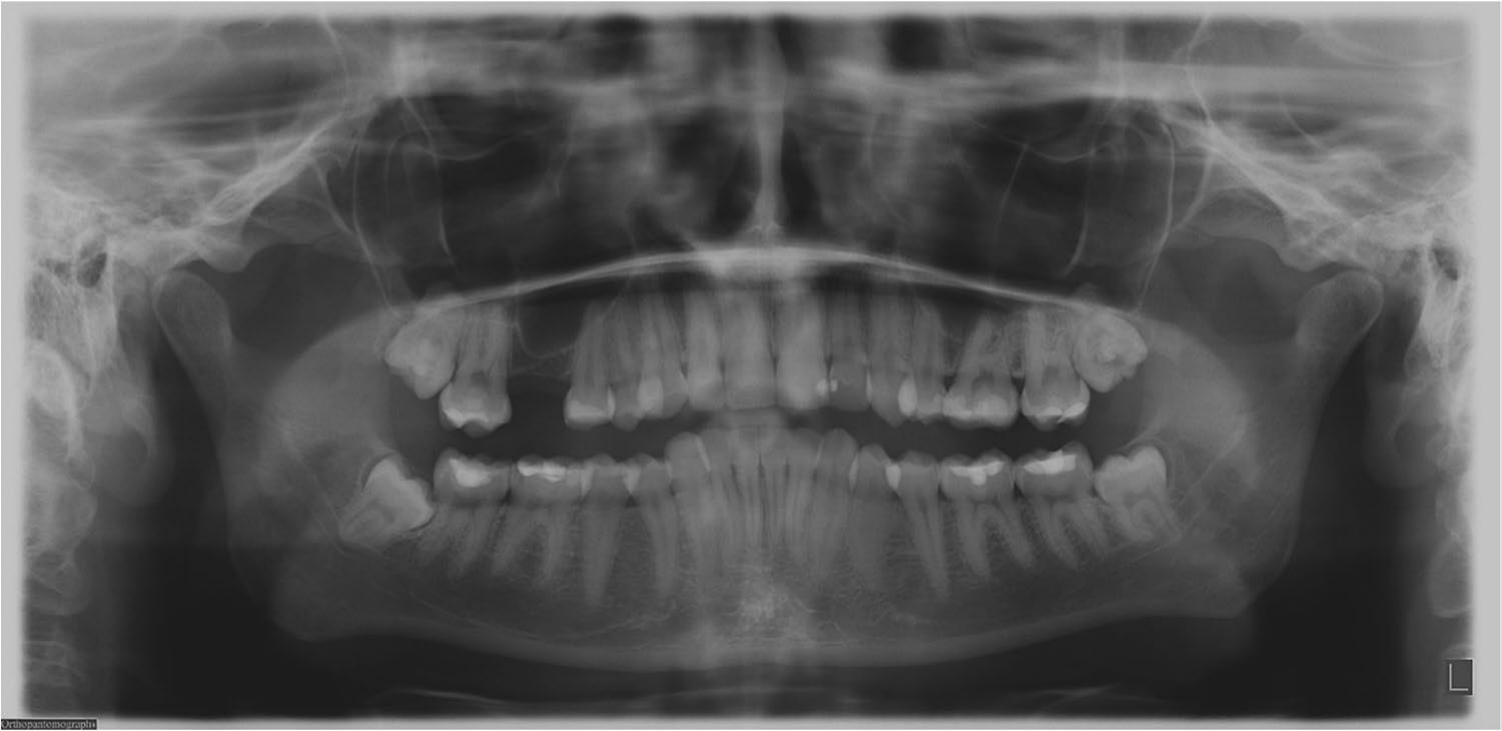
A preoperative panoramic radiograph of a 27-year-old female patient before the coronectomy of the right mandibular third molar

**Fig. 8:**
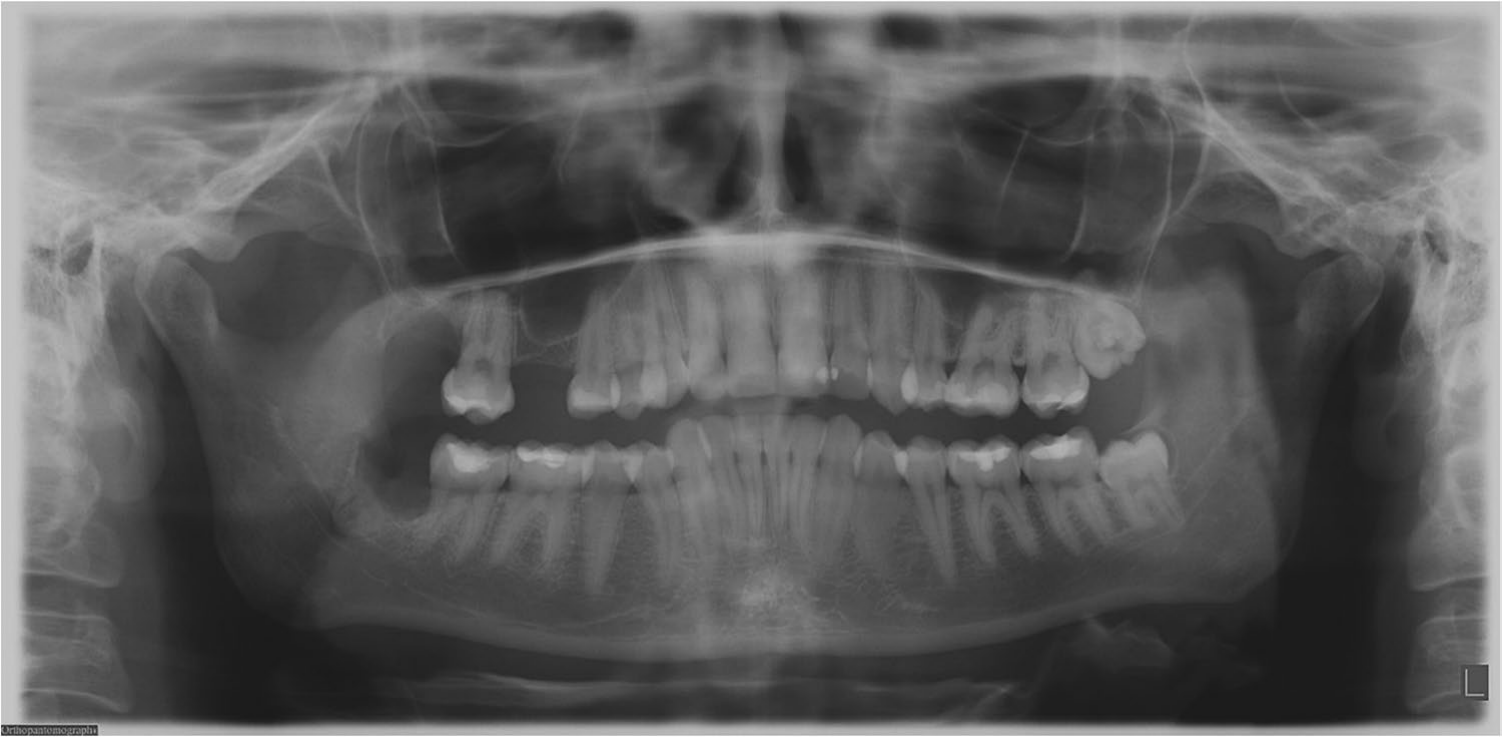
Panoramic radiograph the same patient, 2 months after coronectomy of the right mandibular third molar and removal of the right maxillary third molar

**Fig. 9:**
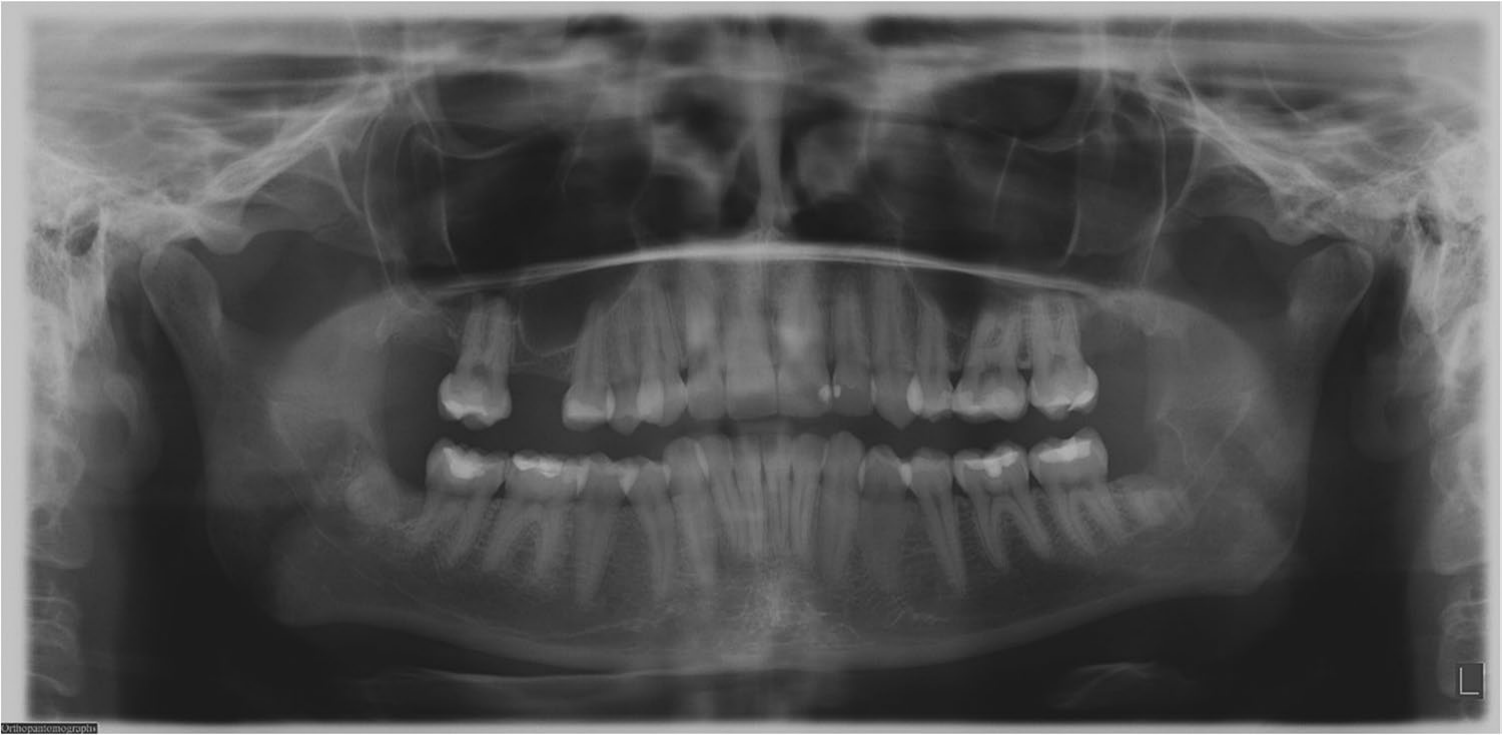
Panoramic radiograph of the same patient performed 6 months after coronectomy of the right mandibular third molar and coronectomy of the left mandibular third molar and removal of the left maxillary third molar

Figures 7–9 illustrate the preoperative panoramic radiograph of a 27-year-old female patient before the coronectomy of the right mandibular third molar (Fig. 7), at the 2-month follow-up (Fig. 8) and at the 6-month follow-up (Fig. 9).

For this study, we performed 165 coronectomies in 141 patients (96 females and 45 males; mean age 33.1 years, SD 16.0). Out of 141 patients, 121 patients appeared for the 2-month check-up (83 females and 38 males; mean age 32.8 years, SD 15.2) and 141 coronectomies were performed. The 6-month check-up was completed by 73 patients (47 females and 26 males; mean age 34.3 years, SD 16.1), and 80 coronectomies were performed.

Two cases of postoperative infection occurred during the first postoperative week. After 3 days, an abscess appeared and was drained, followed by a 5-day course of amoxicillin 500 mg 3 times a day. Postoperative alveolitis did not occur in any of the patients.

Three patients were excluded from the main study group, due to the removal of the mesial or distal root during surgery, making it impossible to preform either of the measuring techniques used for the main study group. Two patients that attended the 6-month check-up appointment did not attend the 2-month check-up appointment. They were excluded from the 0–2- and 2–6-month analyses; they were still included in the 0–6-month analyses. Another three patients did not have a second molar and were therefore excluded from the impaction pattern analysis.

### Fused and tangent root measurements (main study group; n = 138)

In the main study group, the measurements for all third molars with a line tangent to two separate apices and all merged root measurements were measured. The mean total root migrations at 2 months (n = 138) and 6 months (n = 78) postoperatively were 3.30 mm (SD 2.53 mm) and 5.27 mm (SD 3.14 mm), respectively. The mean root migration in the 2–6-month interval (n = 76) was 2.58 mm (SD 2.07 mm).

The mean root migration at 6 months postoperatively was significantly greater in female patients (6.00 mm, SD 3.34 mm) than in male patients (3.78, SD 2.14; *p* = *0.002*). Age was significantly related to the extent of root migration. Patients aged 18–21 years (n = 23), 22–25 years (n = 39), and 26–30 years (n = 32) showed greater migration than patients aged 40 + years (n = 18; *p* = *0.026, p* = *0.012,* and *p* = *0.02*, respectively) at 6 months postoperatively.

The extent of root migration was not significantly affected by the impaction depth, inclination, or the space between the distal part of the second molar and the ramus of the mandible. Tables 1, 2, and 3 provide an overview of the mean root migration for the 0–2-month interval (Table 1), the 0–6-month interval (Table 2), and the 2–6-month interval (Table 3).

**Table 1:** Mean root migration for the main study group, observed 0–2 months

Characteristics for the indicated time interval 0–2-month interval	N	Mean root migration ± SD, mm		P-value
Coronectomies performed	138			
Sex*				*0.002*
Female	81	3.34 ± 2.67		
Male	37	2.06 ± 1.46		
Mean age	32.6 ± 14.9 years			
Impaction depth				0,092
A	27	4.19 ± 3.07		
B	103	3.21 ± 2.35		
C	5	1.98 ± 1.82		
Space between the second molar and ramus				0.765
I	29	3.60 ± 2.89		
II	74	3.22 ± 2.32		
III	35	3.21 ± 2.70		
Inclination				0.205
Mesial	97	3.46 ± 2.57		
Distal	19	2.34 ± 2.04		
Vertical	22	3.39 ± 2.67		
Age group (comparison to 40 + y)				*0.003*
18–21*	23	4.34 ± 2.31		*0.000*
22–25*	39	3.47 ± 2.44		*0.007*
26–30*	32	3.88 ± 2.72		*0.002*
31–40	23	2.65 ± 2.58		0.161
40 +	18	1.58 ± 1.57		reference

^*^P < 0.05; Impaction depth: A: third molar (M3) height above the second molar (M2); B: M3 height shorter than M2; C all M3 below the cementoenamel junction of M2; space between M2 and ramus: I: ≥ mesio-distal diameter of M3; II: < mesio-distal diameter of M3; III: no space: all M3 in the mandible

**Table 2:** Mean root migration for the main study group, observed 0–6 months

Characteristics for the indicated time interval 0–6-month interval	n	Mean root migration ± SD, mm	P-value
Coronectomies performed	78		
Sex*			*0.002*
Female	44	6.00 ± 3.34	
Male	27	3.78 ± 2.14	
Mean age	33.9 ± 15.6		
Impaction depth			0.359
A	16	5.98 ± 3.23	
B	57	5.29 ± 3.05	
C	2	2.77 ± 2.94	
Space between the second molar and ramus			0.808
I	12	5.44 ± 3.24	
II	44	5.40 ± 2.91	
III	22	4.89 ± 3.62	
Inclination			0.143
Mesial	55	5.00 ± 3.23	
Distal	12	4.89 ± 2.31	
Vertical	11	6.99 ± 3.14	
Age group, y (compared to 40 + y)			*0.008*
18–21*	11	6.10 ± 3.14	*0.026*
22–25*	25	5.96 ± 2.96	*0.012*
26–30*	10	7.42 ± 3.23	*0.002*
31–40	17	4.23 ± 2.55	0.410
40 +	12	3.31 ± 2.99	reference

^*^P < 0.05; impaction depth: A: third molar (M3) height above the second molar (M2); B: M3 height shorter than M2; C all M3 below the cementoenamel junction of M2; space between M2 and ramus: I: ≥ mesio-distal diameter of M3; II: < mesio-distal diameter of M3; III: no space: all M3 in the mandible

**Table 3:** Mean root migration for the main study group, observed in the 2–6-month interval

Characteristics for the indicated time interval 2–6-month interval	n	Mean root migration ± SD, mm	P-value
Coronectomies performed	76		
Sex*			*0.002*
Female	42	2.94 ± 2.23	
Male	27	1.97 ± 1.63	
Mean age	34.2 ± 15.7 years		
Impaction depth			0.727
A	15	2.41 ± 1.81	
B	56	2.67 ± 2.08	
C	2	1.66 ± 1.04	
Space between the second molar and ramus			0.512
I	12	2.32 ± 1.95	
II	43	2.44 ± 1.78	
III	21	3.03 ± 2.64	
Inclination			0.698
Mesial	54	2.52 ± 2.17	
Distal	12	3.04 ± 2.00	
Vertical	10	2.37 ± 1.62	
Age group			0.097
18–21*	11	2.43 ± 2.56	
22–25*	23	2.57 ± 1.76	
26–30*	10	4.22 ± 2.73	
31–40	17	2.05 ± 1.48	
40 +	12	2.58 ± 2.10	

*P < 0.05; Impaction depth: A: third molar (M3) height above the second molar (M2); B: M3 height shorter than M2; C all M3 below the cementoenamel junction of M2; space between M2 and ramus: I: ≥ mesio-distal diameter of M3; II: < mesio-distal diameter of M3; III: no space: all M3 in the mandible

### Mesial root measurement study group (n = 76)

When only the mesial roots were measured, the mean total root migration distances at 2 months (n = 76) and 6 months (n = 42) postoperatively were 3.62 mm (SD 2.85 mm) and 5.19 mm (SD 3.00 mm), respectively. The mean root migration in the 2–6-month (n = 42) interval was 2.76 mm (SD 2.68 mm).

Age was significantly related to the extent of root migration in the first two postoperative months; ages 18–21 years showed greater migration than ages 40 + years (*p* = *0.028*). In the first 2 months, the impaction depth also affected the extent of root migration: group A showed significantly greater migration than group B (p < 0.001).

Among patients in the mesial study group, there were no cases with a category C impaction depth. The impaction space affected the extent of migration in the 2–6-month interval. Group I showed significantly greater migration than group III (*p* = *0.017*). However, the third molar inclination and patient sex did not significantly affect the extent of root migration in this study group.

### Distal root measurement study group (n = 75)

When only the distal roots were measured, the mean total root migrations at 2 months (n = 75) and 6 months (n = 39) postoperatively were 3.74 mm (SD 2.54 mm) and 5.34 mm (SD 3.21 mm), respectively. The mean root migration in the 2–6-month (n = 39) interval was 2.51 mm (SD 1.51 mm). Age was significantly related to the extent of root migration in the first 2 months postoperatively. Patients aged 18–25 years showed significantly greater migration than patients aged 40 + years (*p* = *0,049, p* = *0,023*).

In the first 2 months, the impaction depth also affected the extent of root migration. Group A showed significantly greater migration than groups B and C (*p* = *0.002*). Additionally, in the 0–6-month interval, group A showed significantly greater migration than groups B and C (*0.046*). In the 2–6-month interval, vertically impacted roots showed less migration than mesially inclined (*p* = *0.036*) and distally inclined (*p* = *0.01*) roots. The impaction space also affected the extent of root migration in the 2–6-month interval. Group I showed significantly less migration than groups II (*p* = *0.019*) and III (*p* = *0.003*). Patient sex did not show a significant effect on the extent of root migration in this study group.

## Discussion

This study investigated the migration of the third molar root complex in the first 6 months after a mandibular third molar coronectomy. We examined patients at 2 and 6 months postoperatively. We found that, in the main group (n = 138), the mean root migrations at 2 and 6 months were: 3.30 mm (SD 2.53 mm) and 5.27 mm (SD 3.14 mm), respectively. In the 2–6-month interval, the mean root migration was 2.58 mm (SD 2.07 mm). The extents of migration were similar in the 0–2-month interval and the 2–6-month interval (*p* = *0.529*), which suggested that the root complex migrated faster in the first two months postoperatively. Age and patient sex both affected the extent of root migration. A younger age was associated with greater root migration, and female patients showed significantly greater migration than male patients (*p* = *0.002*).

Previous studies mostly focused on analyzing long-term migration after a coronectomy, but these studies did not consider migration at 2 months postoperatively. Some studies have assessed root migration at 3 months postoperatively and found a mean root migration of 1.84–2.8 mm [8, 13, 17, 18]. In the present study, the mean root migration at 2 months was 3.30 mm, which was relatively high. A potential explanation for this high value could be that our female/male ratio was relatively high, compared to previous studies, which could have led to a higher mean root migration. We found a mean root migration ratio for females of 6.00 mm and 3.78 mm for males.

The root migration at 6 months has been widely investigated. In previous studies, the mean root migrations at 6 months postoperatively ranged from 1.9 mm to 4.64 mm [2, 7, 11, 18–20]. In the present study, the mean root migration at 6 months was 5.27 mm, which again was relatively high. As previously described, we found no difference between the mean root migrations during the 0–2-month interval and the 2–6-month interval. This finding suggested that the roots migrated rapidly in the first 2 months postoperatively, and the pace of migration slowed by half in the 2–6-month interval. Kohara et al. [13] and Leung [22] assessed the root migration at 3 months postoperatively and found that the roots migrated the most, and thus fastest, in the 0–3-month interval. Other studies have reported that root migration rate, and therefore also the pace, was highest in the first postoperative intervals, typically 0–6 or 0–12 months [2, 6, 11, 12]. The findings from the studies by Kohara et al. [13] and Leung [22] supported our finding that the pace of migration was highest in the first 2 months postoperatively, instead of during the 6–12-month interval, as previously thought.

The effect of patient age on the extent of migration has been researched widely. The most common finding was that older age was negatively correlated with the extent of root migration. For example, Goto et al. [21] found a significant difference between the extent of migration between patients in their 20 s and patients ≥ 30 years old. Yeung et al. [14] also found that increasing age was negatively correlated with the extent of root migration. The results of this study suggest that both patient sex and age significantly affect root migration in the main study group. Older age was negatively correlated with the extent of root migration. Patients aged 18–30 years (means 5.96 to 7.42 mm) showed significantly greater migration than patients aged 40 + years (mean 3.31 mm). Furthermore, patients aged 26–30 years showed significantly greater migration (mean 7.42 mm) than patients aged 31–40 years (mean 4.23 mm). The results of the present study were consistent with those of previous studies.

The effect of sex on the extent of migration has also been widely assessed in the literature, but results have been inconsistent. Leung and Cheung [2], Kohara et al. [13], and Goto et al. [21] found greater migration in female patients than in male patients. However, Leung and Cheung [11], Pedersen et al. [12], Yeung et al. [14], and Yan et al. [19] did not find a significant effect of sex on the extent of root migration. In this study, we found a significantly greater migration (mean 6.00 mm) for female patients than male patients (mean 3.78 mm). Further research is necessary to determine whether sex influences root migration.

In the present study, root migration was measured in three different ways. In the literature, different methods were used for measuring root migration on an X-OPT image for third molars with one root. We noticed that there was a lack of measuring techniques for third molars with more than one root on an X-OPT image. Consequently, we decided to measure migration for the mesial and distal roots separately. The measuring technique used in this study was fairly similar to the technique used by Leung and Cheung [8]; they also measured root migration as the distance between the point at which the upper white line of the inferior alveolar canal intercepted the long axis of the molar and the point at which the apex of the root intercepted the long axis of the molar. In their study, no technique was specified for third molars with two separate roots. Alternatively, Kohara et al. [13] and Monaco et al. [15] used the distal part of the second molar as a reference point to measure root migration. That technique was limited, because the second molar is not a very consistent reference point; it can be affected by processes in the oral cavity, like decay or erosive tooth wear. Thus, that measurement technique was less reliable than techniques that use the inferior alveolar canal as reference. However, the benefit of both those measuring techniques was that the same method could be used for third molars with fused roots and third molars with two separate roots.

Our results for the separate mesial and distal root measurements differed from the measurements obtained in the main study group. In the main study group, the impaction pattern did not significantly affect the extent of root migration. In contrast, in the mesial and distal study groups, the impaction pattern did affect the extent of root migration.

In the distal root measurements, roots that were distally inclined showed less migration in the 2–6-month interval than roots that were mesially or vertically inclined. In the 0–2-month and 0–6-month intervals, roots with shallow impaction depths (category A) showed significantly more migration than roots with intermediate impaction depths (category B).

In the mesial measurement study group, during the 0–2-month interval, the roots in category A also showed significantly greater migration than the roots in category B. In the 2–6-month and 0–6-month intervals, the roots with no spacing (category III, almost all the third molar in the mandible) showed significantly greater migration than roots with sufficient space (category I, space between the ramus and the distal part of the second molar could accommodate the mesio-distal diameter of the third molar).

A study by Yan et al. [19] reported that the impaction depth, root angulation, and retromolar space all affected root migration from the apex to the crown. Kouwenberg et al. [5] found that impaction depth significantly affected root migration. Third molars impacted below the cervical margin of the second molar showed significantly less migration than third molars impacted between the occlusal plane and the cervical margin of the second molar. Other studies have reported that the impaction pattern did not affect the extent of root migration [2, 11, 14, 21]. Further research is needed to determine whether the impaction pattern affects root migration.

Root migration in both the mesial and distal study groups was affected by age. The youngest patients showed significantly greater migration than the oldest patients. However, patient sex did not significantly affect the extent of root migration in either the mesial or distal study group.

This study had several limitations. First, there was a great loss to follow-up; thus, the sample size was smaller than expected. Of the 141 patients that underwent 165 coronectomies, only 121 patients completed the 2-month follow-up, resulting in 141 coronectomies. Only 73 patients that underwent 80 coronectomies completed the 6-month follow-up. This high dropout rate was partly due to the COVID-19 pandemic, which led to many appointment cancellations. Other patients simply did not attend the appointments.

Another limitation of this study was the use of X-OPT images. X-OPT images only show two dimensions. Consequently, migration could only be measured in the direction from the apex to the crown. Any potential rotations and migrations in the buccal-to-lingual direction could not be measured on the X-OPT images; thus, the lack of three-dimensional measurements might have distorted our results. To eliminate this limitation, cone-beam computed tomography (CBCT) could be used to assess root migration in three dimensions and that would provide a better representation of reality. Yeung et al. [14] investigated root migration and rotation at 4 to 8.5 years postoperatively with the use of CBCT. Similar to the present study, they measured root migration in a (simulated) panoramic view. In addition, they recorded horizontal, vertical, and transverse translations and relative rotations. Remarkably, they found that only age significantly affected the extent of root migration. Patient sex did not affect root migration, translation, or rotation. In contrast, the present study showed that both age and sex affected root migration. However, age, root form, and eruption status could all significantly affect postoperative translations and rotations.

When focusing on clinical relevance, the question arises as to whether it is really necessary to know the exact extent and direction of root migration after a mandibular third molar coronectomy. The main complication of root migration is the eruption of the roots into the oral cavity. When this happens, the patient must undergo a repeat operation in order to remove the remaining root complex. To predict which patients might be susceptible to eruption, it is important to know the type and extent of migration, the factors that affect the extent of root migration, and when root migration stops. With this information, we can form a general profile of patients that are at high risk of root eruption. Moreover, a sufficient follow-up schedule could be arranged.

In conclusion, this study showed that the pace of migration was highest during the first two postoperative months. Age and sex both affected the extent of root migration within the first 6 postoperative months. Increasing age was negatively correlated with the extent of root migration, and female patients showed significantly greater migration than male patients. More studies are needed to determine whether the pattern of impaction affects the extent of root migration. Future studies should assess root migration in greater detail preferably by using three-dimensional imaging.
