# Correction to: Early root migration after a mandibular third molar coronectomy

**DOI:** 10.1007/s10006-022-01079-6

**Published:** 2022-06-03

**Authors:** Rashida N. Simons, Jacco G. Tuk, Jean-Pierre T. F. Ho, Naichuan Su, Jerome A. Lindeboom

**Affiliations:** 1 Department of Oral and Maxillofacial Surgery, Amsterdam University Medical Center and Amstelland Hospital, University of Amsterdam, Meibergdreef 9, 1105 AZ Amsterdam, the Netherlands; 2 Departments of Oral and Maxillofacial Surgery, Amsterdam University Medical Centers and Northwest Clinics, University of Amsterdam, Amsterdam, the Netherlands; 3 Department of Oral Public Health, Academic Centre for Dentistry Amsterdam (ACTA), University of Amsterdam and Vrije Universiteit Amsterdam, Amsterdam, The Netherlands

**Correction to: Oral and Maxillofacial Surgery (2022)**

10.1007/s10006-022-01072-z

Originally, the article was published with an error in Figure 4 legend. The legend should read:

- **Fig. 4** Measuring technique 1: The distance between the roots and the inferior alveolar canal (white lines). Point A: intersection between the long axis of the molar and the upper white line of the inferior alveolar canal; point B: intersection between the long axis of the molar (red lines) and the apices of the mesial and distal roots

The original article has been corrected.

